# S-ketamine and intranasal application: alternatives for the castration of male suckling piglets?

**DOI:** 10.1186/s12917-021-02826-9

**Published:** 2021-03-16

**Authors:** Sabrina Becker, Anna Maier, Saskia Peters, Kathrin Büttner, Gerald Reiner

**Affiliations:** 1 Department for Clinical Veterinary Sciences, Clinic for Swine, Justus-Liebig-University, Giessen, Germany; 2 Department of Animal Welfare, Justus-Liebig-University, Giessen, Germany; 3 Unit for Biomathematics and Data Processing, Faculty of Veterinary Medicine, Justus Liebig University, Frankfurter Str. 95, 35392 Giessen, Germany

**Keywords:** Swine, Piglet castration, Ketamine

## Abstract

**Background:**

The intramuscular injection of ketamine and azaperone was proposed as a suitable anaesthesia for male suckling piglets for surgical castration. However, this can be opposed by massive defensive movements, hypothermia and tachycardia during castration and a long recovery period. The aim of the present study was to test whether the use of S-ketamine and/or a change in the route of application from intramuscular to intranasal could reduce stress responses and the duration of recovery compared to the intramuscular route and the use of racemic ketamine. Seventy-eight healthy, five-day-old male piglets were randomized to six treatment groups in a blinded experimental study, matched by litter and weight. Experimental groups were A (15 mg kg-1 S-ketamine + 2 mg kg-1 azaperone, i.m., surgical castration), B (15 mg kg-1 R/S-ketamine racemate + 2 mg kg-1 azaperone, i.m., surgical castration), C (30 mg kg-1 S-ketamine + 2 mg kg-1 azaperone, i.n., surgical castration), D (15 mg kg-1 R/S-ketamine racemate + 2 mg kg-1 azaperone, i.m.; not castrated), E (positive control group; no anesthesia, surgical castration) and F (negative control group; no anesthesia, not castrated).

**Results:**

S-ketamine reduced the defensive movement score during castration to a similar extent to racemic ketamine when administered intramuscularly but not via the intranasal route. However, the effects of S-ketamine (both routes) on the increase in cortisol levels and decrease in body temperature were similar to those induced by racemic ketamine. A reduction of the long recovery time known for ketamine-azaperone anaesthesia could not be achieved with S-ketamine in the given dosage, regardless of the route of application. The intranasal administration of ketamine was difficult with the available formulation as the necessary amount exceeded the capacity of the nose cavity.

**Conclusions:**

Neither the use of S-ketamine nor intranasal administration can be suitable alternatives for the anaesthesia of male suckling piglets for castration.

## Background

The intramuscular (i.m.) injection of ketamine in combination with azaperone was proposed as a practicable approach for the anaesthesia of male piglets in preparation for castration [1]. However, an extended time for the castration process and a longer recovery period were discussed as major disadvantages of this method [2]. Higher losses of anaesthetised piglets during the recovery phase due to injuries by the sow and hypothermia compared to non-anaesthetised piglets were described by Kmiec et al. [3]. A long recovery time can also lead to the loss of suckling periods, with possible disadvantages for the development of the affected animals [4].

Ketamine, as racemate (same mixture of S (+) and R (-) isomers) is the only preparation authorised for injection anaesthesia in pigs. It leads to sedation, analgesia and immobility [5]. For general anaesthesia, ketamine must be combined with a neuroleptic (azaperone) to compensate muscle hypertension [6]. Azaperone is the only approved neuroleptic for pigs [6].

The two enantiomers of ketamine have different properties such as binding affinity to receptors. Hustveit et al. [7] investigated the binding affinity of R- and S- ketamine to the specific receptors in a guinea pig ileum model. It is generally considered that supraspinal blockade of the NMDA receptor is responsible for the most important anti-nociceptive effects of ketamine. However, ketamine shows also affinity for the different opioid receptors. The S-enantiomer seems to have a stronger analgesic effect than the R-enantiomer due to its three times higher binding affinity to the NMDA receptor, as well as to µ- and k-opioid-receptors [7]. R-ketamine is definitely also associated with hallucinogenic effects, although to a lesser extent compared to S-ketamine. In humans, it is known that the R-enantiomer, in contrast to the S-enantiomer, has a higher affinity to the σ-opioid receptors, which causes hallucinogenic changes of consciousness [8]. This aspect and the binding to muscarinic receptors might be a cause of agitation that can usually be seen in pigs under and after ketamine anaesthesia [7].

The effects of ketamine at the various receptors have not yet been studied in the pig model. However, observations made in pigs under ketamine-induced anaesthesia have confirmed the typical hallucinogenic symptoms in the recovery phase [9]. Additionally, Schmidt et al. [10] found less pronounced haemodynamic alterations of S-ketamine in pigs, when compared to the RS-racemate.

Evidence of a reduced hallucinogenic effect of S-racemate, reduced hemodynamic alterations, a somewhat shorter recovery phase and a similar anaesthetic impact in 9-week-old pigs weighing 25 kg [6] made it appear reasonable to study the effect of S-racemate on suckling piglets at the typical castration age of 5 days and a weight of 2 kg. A second aspect is that Ketamine is applied intramuscularly (i.m.) or intravenously (i.v.). The injection of the acidic substance can lead to additional pain and damage and the anaesthesia is imprecisely controlled; a potential factor for the prolonged recovery period. Intranasal application of pharmaceuticals is already established in human and veterinary medicine. The mucosa of the nose provides a huge resorptive surface and permeability; while irritations or painful injections are omitted [11, 12]. In addition, the blood-brain barrier is circumvented with the intranasal application. During this procedure, the drugs reach the brain via the olfactory or trigeminal nerve, which innervates the nasal cavity [13]. Initially, the olfactory nerve was thought to be the main part of this pathway [14–17]. However, recent findings show that the trigeminal nerve is also involved, especially in the caudal brain regions and the spinal cord [18, 19].

Because of the high permeability and the close proximity to the central nervous system (CNS), there is a quick onset of the effects, comparable to intravenous injections [11, 12, 20]. Administration is no more difficult than an injection and accidental injuries are excluded [20–22]. Intranasal application of ketamine (15 mg kg^− 1^), azaperone (1 mg kg^− 1^) and climazolam (1.5 mg kg ^− 1^) was tested in pigs by Axiak et al. [5] and its basic effectiveness was proven, although the applied dosage was too low. Compared to i.m. injection, the i.n. group showed significantly stronger defensive movements, but also a shortened recovery time. These results point towards an interesting approach for intranasal application of ketamine in suckling piglets during the first week of life. However, an intranasal application of ketamine only makes sense if an effect comparable to intramuscular application can be achieved. It is questionable whether the application of a higher dose of ketamine intranasally is possible and whether the advantages of a shorter recovery phase compared to the RS-racemate can still be achieved under these conditions.

The present study aims to combine the approaches of Bettschart-Wolfensberger et al. [6] on the use of S-ketamine and of Axiak et al. [5] on the intranasal application of ketamine in suckling piglets during their first week of life. We tested the hypothesis that the use of S-ketamine and the intranasal application are superior compared to the intramuscular application of the RS-racemate of ketamine for suckling piglet castration. Additionally, the drugs butorphanol (Bettschart-Wolfensberger et al. [6]) and Climazolam (Axiak et al. [5]) were excluded from the present study as both are not approved for food-producing animals in most countries.

## Results

**Fig. 1:**
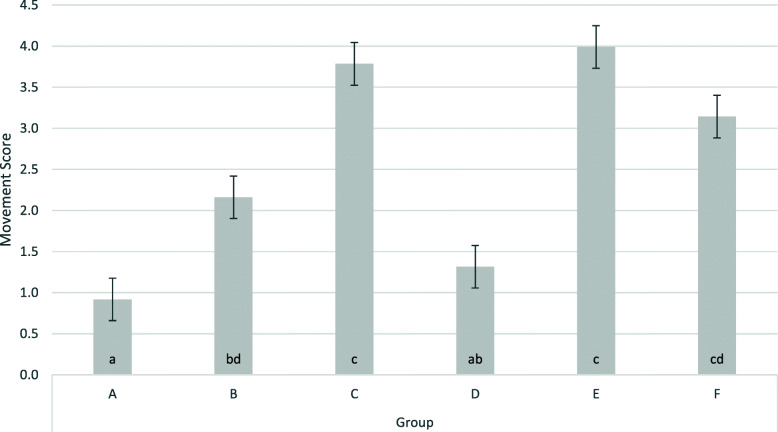
Movement scores during castration/sham castration by trial group. The figure shows least square means and standard errors; **a**: S-ketamine, i.m., surgical castration; **b**: R/S-ketamine, i.m., surgical castration; **c**: S-ketamine; i.n., surgical castration; **d**: R/S-ketamine, i.m., sham castration; **e**: no anaesthesia, surgical castration; **f**: no anaesthesia, sham castration; i.m.: intra muscular application; i.n. intra nasal application; groups with the same lower case were not significantly different (*P* < 0.05); ketamine always administered together with azaperone; all piglets received the NSAID Flunixine

The piglets weighed 1.9 ± 0.49 kg. The movement score at the time of castration (Table 1; Fig. 1) was significantly increased at *p* < 0.1 in the castrated and not anaesthesized (positive) control group E as compared to the group without anaesthesia and without castration (group F). R/S-ketamine and azaperone (group B) reduced the movement score significantly compared to group E. The replacement of the R/S-racemate by the pure S-racemate (group A) significantly further decreased the observed movement score. This score was more influenced in castrated piglets (group A) than in sham castrated piglets (group D). Changing the location of application from i.m. (group A and B) to i.n. (group C) did not improve the original situation without anaesthesia (group E).

**Table 1:** The movement score during treatment according to Axiac et al. [5]

Score	Reaction
0	No reaction or vocalization
1	Movement of one limb, no vocalization
2	Movement of two limbs, no vocalization
3	Movement of all limbs with or without vocalization
4	Strong movements of all limbs and vocalization

**Fig. 2:**
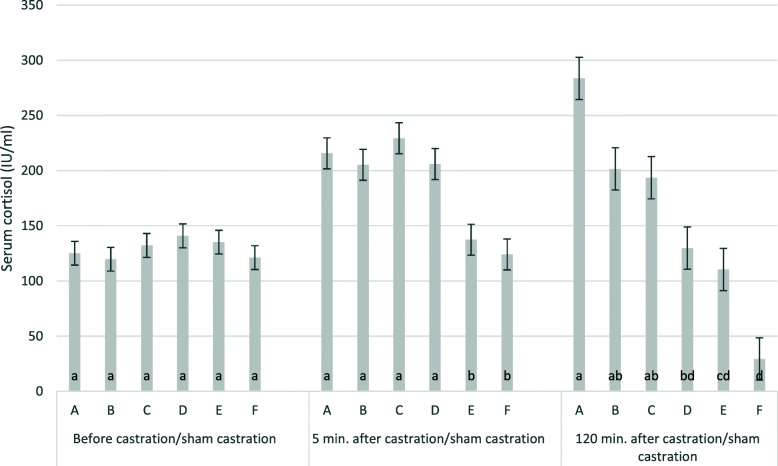
Cortisol levels 5 and 120 min. after castration/sham castration. The figure shows least square means and standard errors for cortisol levels (IU/ml) min.; **a**: S-ketamine, i.m., surgical castration; **b**: R/S-ketamine, i.m., surgical castration; **c**: S-ketamine; i.n., surgical castration; **d**: R/S-ketamine, i.m., sham castration; **e**: no anaesthesia, surgical castration; **f**: no anaesthesia, sham castration; i.m.: intra muscular application; i.n. intra nasal application; groups with different lower cases were significantly different; ketamine always administered together with azaperone; all piglets received the NSAID Flunixine

Mean baseline cortisol levels (Table 2) were 129 ± 4.3 IU/ml. Effects of time and treatment were significant. Five minutes and one hundred twenty minutes after castration, the average cortisol levels reached 186 ± 5.7 and 158 ± 7.8 IU, respectively. The effect of the time in relation to castration/sham castration was highly significant. Cortisol levels did not differ between groups before castration/sham castration but the treatment had a highly significant effect on serum cortisol levels five and 120 min. after castration/sham castration. With castration under ketamine anaesthesia (A, B, C), serum cortisol levels were higher 5 and 120 min. after castration than before. The highest increase was achieved in group A (S-ketamine i.m.). Cortisol levels decreased significantly in the not castrated piglets (group F) 120 min. after the handling procedure (Table 2; Fig. 2). Cortisol levels increased significantly from 5-120 min. (215 to 283 IU/ml) in group A, but decreased from 5-120 min. in groups C to F. The intranasal treated group (C) had the highest cortisol slope five min. after castration. Five min. after castration, the anaesthetised groups (A to C) showed significantly higher cortisol levels compared to both control groups (E, F). Even in the sham castrated groups, serum cortisol levels decreased significantly less after five and 120 min. with ketamine (D) than without ketamine (F). The effect of the group, 5 and 120 min. after castration/sham castration was statistically significant with *P* < 0.001, with coefficients of determination (R^2^) of 0.39 and 0.56, respectively.

**Table 2:** Least square means and standard errors of clinical parameters before and 5 and 120 min. after castration/sham castration by treatment group

Clinical parameter	Time point in relation to castration/sham castration	Total	Group						
			A	B	C	D	E	F	*P* (group)
Serum cortisol (IU/ml)	Before	129 ± 4.3	125 ± 10.7	119 ± 10.7	132 ± 10.7	140 ± 10.7	135 ± 10.7	121 ± 10.7	n.s.
	5 min. after	186 ± 5.7	215 ± 14.0	205 ± 14.0	229 ± 14.0	205 ± 14.0	137 ± 14.0	123 ± 14.0	<0.001
	120 min. after	158 ± 7.8	283 ± 19.1	201 ± 19.1	193 ± 19.1	129 ± 19.1	110 ± 19.1	29 ± 19.1	<0.001
	P	<0.001	<0.001	<0.001	<0.001	0.017	n.s.	<0.001	
Hearth rate (/min)	Before	150 ± 3.0	150 ± 7.3	148 ± 7.3	152 ± 7.3	145 ± 7.3	150 ± 7.3	154 ± 7.3	n.s.
	5 min. after	140 ± 4.4	128 ± 10.9	125 ± 10.9	137 ± 10.9	132 ± 10.9	153 ± 10.9	164 ± 10.9	n.s.
	120 min. after	157 ± 4.1	150 ± 10.1	172 ± 10.1	153 ± 10.1	138 ± 10.1	157 ± 10.1	171 ± 10.1	n.s.
	P	0.012	n.s.	0.021	n.s.	n.s.	n.s.	n.s.	
Breaths per min.	Before	75 ± 2.1	76 ± 5.2	76 ± 5.2	70 ± 5.2	72 ± 5.2	83 ± 5.2	70 ± 5.2	n.s.
	5 min. after	72 ± 1.8	66 ± 4.4	72 ± 4.4	64 ± 4.4	81 ± 4.4	73 ± 4.4	71 ± 4.4	n.s.
	120 min. after	74 ± 3.1	72 ± 7.7	81 ± 7.7	66 ± 7.7	78 ± 7.7	70 ± 7.7	74 ± 7.7	n.s.
	P	n.s.	n.s.	n.s.	n.s.	n.s.	n.s.	n.s.	
Rectal temperature (°C)	Before	39.4 ± 0.51	39.7 ± 0.12	39.5 ± 0.12	39.0 ± 0.12	39.3 ± 0.12	39.5 ± 0.12	39.2 ± 0.12	n.s.
	5 min. after	38.2 ± 0.93	37.7 ± 0.23	37.9 ± 0.23	38.1 ± 0.23	37.7 ± 0.23	39.0 ± 0.23	38.9 ± 0.23	<0.001
	120 min. after	39.2 ± 0.12	39.0 ± 0.29	39.6 ± 0.29	38.7 ± 0.29	39.2 ± 0.29	39.1 ± 0.29	39.1 ± 0.29	n.s.
	P	<0.001	<0.001	<0.001	0.008	<0.001	n.s.	n.s.	

A: S-ketamine, i.m., surgical castration; B: R/S-ketamine, i.m., surgical castration; C: S-ketamine; i.n., surgical castration; D: R/S-ketamine, i.m., sham castration; E: no anaesthesia, surgical castration; F: no anaesthesia, sham castration; i.m.: intra muscular application; i.n. intra nasal application; ketamine always administered together with azaperone; all piglets received the NSAID Flunixine

*n.s.* not significant

**Fig. 3:**
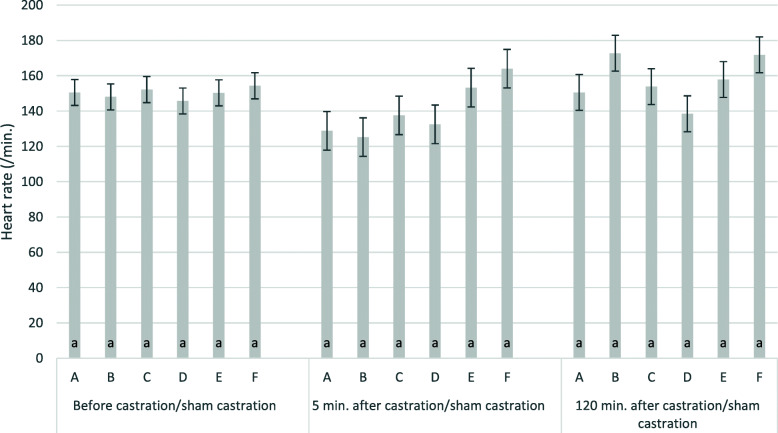
Heart rates 5 and 120 min. after castration/sham castration by group. The figure shows least square means and standard errors for heart rates (/min) min.; **a**: S-ketamine, i.m., surgical castration; **b**: R/S-ketamine, i.m., surgical castration; **c**: S-ketamine; i.n., surgical castration; **d**: R/S-ketamine, i.m., sham castration; **e**: no anaesthesia, surgical castration; **f**: no anaesthesia, sham castration; i.m.: intra muscular application; i.n. intra nasal application; groups with different lower cases were significantly different; ketamine always administered together with azaperone; all piglets received the NSAID Flunixine

Mean baseline heart rates were 150 ± 3 beats per minute. Effects of time point and treatment were not statistically significant. Heart rates 5 and 120 min. after treatment were 140 ± 4.4 beats per minute and 157 ± 4.1 beats per minute, respectively. There was a small tendency for reduced heart rates in ketamine treated groups five min. after castration (Fig. 3, groups A, B, C, D). However, differences between groups were not statistically significant. Explained variance (R^2^) five and 120 min. after castration/sham castration was 24 % and 19 %, respectively.

**Fig. 4:**
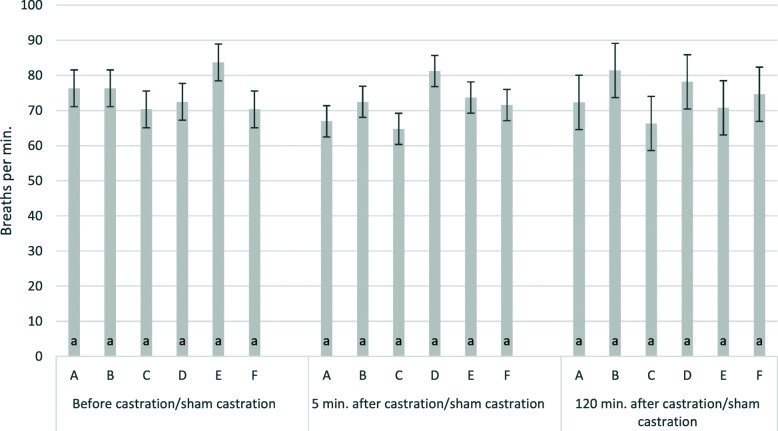
Breaths 5 and 120 min. after castration/sham castration by group. The figure shows least square means and standard errors for breathing rates per min.; **a**: S-ketamine, i.m., surgical castration; **b**: R/S-ketamine, i.m., surgical castration; **c**: S-ketamine; i.n., surgical castration; **d**: R/S-ketamine, i.m., sham castration; **e**: no anaesthesia, surgical castration; **f**: no anaesthesia, sham castration; i.m.: intra muscular application; i.n. intra nasal application; groups with different lower cases were significantly different; ketamine always administered together with azaperone; all piglets received the NSAID Flunixine.i.m.: intra muscular application; i.n.: intra nasal application; values with the same lower case are not significantly different

Mean baseline breathing frequencies before the start of the treatment varied clearly between piglets with 75 ± 2 breaths per minute, but were not significantly different between time points and treatment groups. Frequencies did not significantly change five and 120 min. after handling and treatment (Table 2; Fig. 4). Explained variance (R^2^) 5 and 120 min. after castration/sham castration was 47 % and 25 %, respectively.

**Fig. 5:**
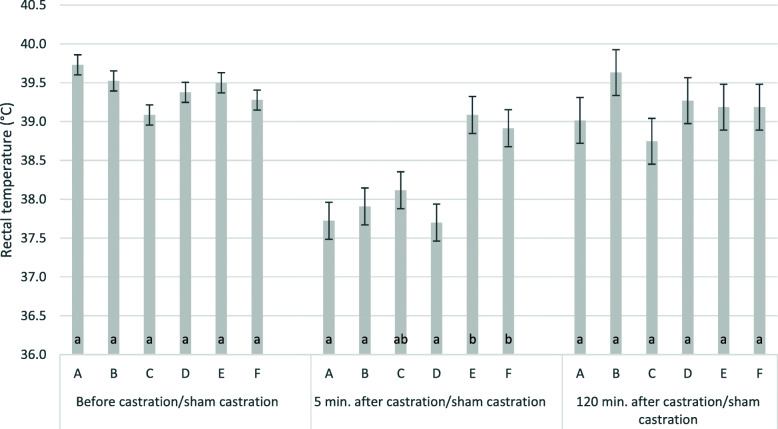
Rectal temperature before, 5 and 120 min. after castration/sham castration by group. The figure shows least square means and standard errors for the rectal temperatures (°C) min.; **a**: S-ketamine, i.m., surgical castration; **b**: R/S-ketamine, i.m., surgical castration; **c**: S-ketamine; i.n., surgical castration; **d**: R/S-ketamine, i.m., sham castration; **e**: no anaesthesia, surgical castration; **f**: no anaesthesia, sham castration; i.m.: intra muscular application; i.n. intra nasal application; groups within the same time with different lower cases were significantly different; ketamine always administered together with azaperone; all piglets received the NSAID Flunixine

Mean body temperature (Table 2) before castration was 39.4 ± 0.5 °C. Five and one hundred twenty minutes after treatment, the temperatures were 38.2 ± 1 °C and 39.2 ± 1 °C. The time point had a significant effect on the body temperature. The effect of the group was significant five min. after castration/sham castration. All ketamine/azaperone treated piglets (groups A, B, C, D) had significantly lower rectal temperatures 5 min. after castration/sham castration than before castration (*P* < 0.001) (Fig. 5). Ketamine/azaperone treated piglets (group A, B and D) both, castrated or not, had a significantly reduced body temperature five min. after treatment when compared to groups E and F which did not receive ketamine/azaperone (Fig. 5). Rectal temperatures dropped by up to 3.2 °C (lower confidence interval_95_) in anaesthesized piglets. There was no difference between the R/S-racemate and the S-ketamine (group A and B). If ketamine/azaperone were given nasally (group C), there was a tendency, but no significant decrease in temperature, because of the high variation in this group. The model explained 42 % (*P* < 0.01) and 7 % (not significant) of the variance of the rectal temperature 5 and 120 min. after castration/sham castration, respectively. Two hours after castration/sham castration, there were neither differences in rectal temperature between groups nor compared to the time point before castration/sham castration.

**Fig. 6:**
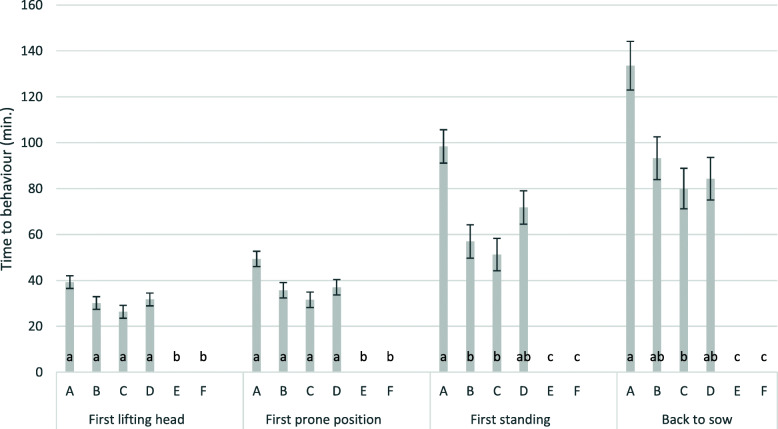
Development of different states of recovery by group. The figure shows least square means and standard errors for the different states of recovery (time in min. until the behaviour occurs); **a**: S-ketamine, i.m., surgical castration; **b**: R/S-ketamine, i.m., surgical castration; **c**: S-ketamine; i.n., surgical castration; **d**: R/S-ketamine, i.m., sham castration; **e**: no anaesthesia, surgical castration; **f**: no anaesthesia, sham castration; i.m.: intra muscular application; i.n. intra nasal application; groups with different lower cases were significantly different; ketamine always administered together with azaperone; all piglets received the NSAID Flunixine

The treatment had a significant effect on the time to first lifting head, first prone position, first standing and on the time to be brought back to sow (*p* < 0.001). Piglets had a prolonged recovery phase (Fig. 6) after ketamine anaesthesia. It took 193 min. until the last piglet was able to walk and was returned to the sow. Piglets could be found in a prone position 31 to 49 min. after treatment. Fifty-one to 98 min. after treatment, piglets were found standing and they were returned to the sow on average 79 to 134 min. after castration. Maximal time spent from castration to lifting head, prone position, standing and removing were 70, 92, 173 and 193 min., respectively. Maximal recovery time was found under i.m. application of S-ketamine (A) (*p* < 0.05), minimum time was found following intra nasal application of S-ketamine (C), which was not significantly different from R/S ketamine i.m. (B). Piglets in group A required longer (*p* < 0.1) to lift their head for the first time and to be in prone position, and they required significantly longer at *p* < 0.05 to stand and to be returned to the sow than piglets of groups B and C. There was no significant difference in the recovery phase between groups B, C and D. Coefficients of determination for the duration from castration/sham castration to first lifting of head, first prone position, first standing and moving the piglet back to sow was 0.69, 0.64, 0.60 and 0.57, respectively.

## Discussion

The present study was conducted based on the hypotheses that the S-enantiomer of ketamine could reduce the response of piglets following the painful stimulus of castration compared to racemic ketamine and that intranasal application could shorten recovery time. The hypothesis was tested based on the movement score (movement of the body and limbs in combination with the piglets’ vocalisation) and the increase of cortisol levels from levels obtained before the treatment (castration or sham castration).

The act of castration after application of an NSAID but without anaesthesia (group E), according to the standard lawful practice at that moment in Germany, increased the average median movement score of the piglets by 25 % when compared to the sham castrated group (F).

S-ketamine at a dosage of 15 mg kg^− 1^ i.m. reduced the movement score at a higher degree than with the racemic ketamine. This effect could however, not be reproduced via the intranasal route. Already the dosage finding, preliminary study revealed that even 30 mg kg^− 1^ S-ketamine i.n. did not reach the effectivity of the intramuscularly applied racemic ketamine, although, with the available formulation, it was the highest dosage that could be applied to the piglets’ nostrils without too much losses. Our findings confirm earlier results, according to which movement scores were reduced by i.m. application of ketamine, although piglets were still able to react on pain stimulation with movements of the limbs [23]. With a reduction of the movement score by 68 %, the racemic ketamine tended to be less effective than the pure S-ketamine (reduction by 78 %). The higher effectivity of S-ketamine compared to the racemic ketamine is in good agreement with results of Bettschart-Wolfensberger et al. [6], who demonstrated that S-ketamine could induce an anaesthesia comparable to R/S ketamine during castration.

Several studies in other animal species demonstrate the benefits of S-Ketamine [24–28]. Shetland ponies receiving S-Ketamine in combination with xylazine got up faster than those administering R/S-Ketamine and xylazine. This is probably due to the faster elimination of S-ketamine and its active metabolite (S-Norketamine) [24]. Comparable results were observed in cats and hamsters [25, 26]. Nevertheless, there is also evidence that the combination of medetomidine and S-ketamine had no advantages in dogs and cats compared to the mixture of the racemate of ketamine and medetomidine [26, 27]. The comparison of the studies is difficult, as the use of ketamine alone is not sufficient for anaesthesia, and a combination with a sedative or neuroleptic is required [6]. Depending on the study, different preparations are used, and their effects should not be underestimated. For example, Larenza et al. [24] described that the heart rate remains the same for both S-ketamine and R/S-ketamine. They conclude that this is due to xylazine, which leads to a reduction of the sympathetic effects of the isoforms. In contrast, a higher heart rate was observed for S-ketamine in combination with isoflurane [28].

However, there are not many studies dealing with S-ketamine in pigs. Bettschart-Wolfensberger et al. [6] worked with a theoretical dosage of 9 mg kg^− 1^ S-ketamine. In the end they applied 13 mg kg^− 1^ after several supplemental applications. Of these 13 mg, 2.25 mg were administered intravenously. Given a higher response to the i.v. application, these 13 mg are comparable to the 15 mg administered i.m. in our study. In further contrast to our study, the study of Bettschart-Wolfensberger et al. [6] additionally used butorphanol that further might be considered as a factor to decrease pain perception and thus, the dosage of S-ketamine. During castration under racemic ketamine anaesthesia, piglets still showed signs of pain [23]. With the aim of reducing the painfulness of castration through the administration of S-ketamine and taking into account the aforementioned aspects of the study of Bettschart-Wolfensberger et al. [6], we decided to work with the same dosage of S-ketamine as for the racemic ketamine. At the same time, S-ketamine at a dosage of 15 mg kg^− 1^ i.m. exacerbated the effects on cortisol levels and the duration of the recovery period. Thus, further studies on S-ketamine for anaesthesia in piglets and pigs are needed to find the optimal dosage.

S-ketamine had no significant effects on the heart and breath rates. Body temperature was reduced in all ketamine-treated groups five min. after castration, as expected, with tendentially lower effects after intranasal application than after administering S-ketamine intramuscularly at a dosage of 15 mg kg^− 1^.

Cortisol levels were significantly increased five min. after castration both, by S-ketamine and the R/S-racemate, regardless of the route of application. Compared to the sham castrated control (group D), cortisol levels were increased 120 min. after ketamine-anaesthesia and castration. Application of S-ketamine at 15 mg kg^− 1^ i.m. showed the strongest effect in this context. Increased cortisol levels can be delayed by up to 20 min. following stress [29, 30]. The present study was conducted under practical conditions and indwelling catheters were not used. Thus, cortisol levels increased in all piglets because of the handling procedure before the beginning of the actual treatment: Piglets were taken away from the sow and put together in a box before the first blood sample was taken for the pre-treatment cortsiol examination. Decreased cortisol levels 120 min. after the treatment in the sham castrated group show that the piglets were able to recover from pre-treatment stress. However, recovery was prevented by castration without anaesthesia and the treatment with ketamine in combination with castration further increased cortisol levels when compared to castration without ketamine. It was shown by others before that ketamine as a NMDA-receptor antagonist promotes the liberation of cortisol by inhibition of the re-uptake of noradrenalin [31–33]. This effect might be more pronounced by S-ketamine at 15 mg kg^− 1^ as compared to the racemic ketamine. It was less pronounced during intranasal application, obviously because of the problem to apply the whole dosage via the intranasal route. This interaction of ketamine with cortisol leads to a significant reduction in the significance of cortisol levels with regard to pain and stress during castration when ketamine is used. Unfortunately, there are currently no practicable methods available that would allow a direct measurement of pain.

One of the biggest problems of ketamine anaesthesia of suckling piglets is the prolonged recovery phase. It was also significantly prolonged by the administration of ketamine in the present study. S-ketamine at a dose of 15 mg kg^− 1^ i.m. had the highest effects, but even the intranasal application of S-ketamine led to effects comparable to the application of racemic ketamine.

Intranasal application was less efficient than the intramuscular. Preliminary tests had demonstrated that the effect of intranasal application of ketamine on the piglets’ movement score during castration increased with 20 to 30 mg ketamine kg^− 1^ bodyweight, but did not reach the efficiency of i.m. application of 15 mg kg^− 1^ ketamine (data not shown). Higher concentrations could not be tested, because of the lack of a higher concentrated ketamine preparation and the impossibility to apply more than 2 ml solution to the piglet’s nostrils. Our results confirm those of Axiak et al. [5] who also found significantly higher movement scores after intranasal application of ketamine as compared to the i.m. route. Additionally, Axiak et al. [5] described higher degrees of tachycardia and tachypnoea when applying ketamine intranasally than after i.m. application.

The lower efficiency and higher variability regarding movement scores and cortisol levels after i.n. application of ketamine was clearly associated with the impossibility to apply the necessary amount of drug to the nasal cavity. Thus, the dosage of the only available S-ketamine preparation was too low for efficient anaesthesia in suckling piglets. Swallowing of parts of the drug followed by delayed uptake by the gastro-intestinal tract as a consequence of too high volumes will lead to an important first pass effect and therefore reduce the amount of active drug reaching the systemic circulation. In humans, it is known that ketamine has a low oral bioavailability [33]. Resorption could also be disrupted by nasal congestion [34]. Axiak et al. [5] described a significant effect of the solution’s temperature. Under 18 °C, they found lower effects, eventually because of vasoconstriction [35]. This effect was excluded in our study, because room temperatures were never below 24.8 °C and ketamine preparations were adapted to room temperature prior to use. Another aspect that could have been responsible for the lower effectiveness of the intranasal application is the duration of the selected period of 15 min. between anaesthesia and castration. We had chosen this route in order to achieve standardisation in accordance with the practice of intramuscular injection and with the methodology of Axiak et al. [5]. Future studies should examine whether extending the exposure time before castration can lead to better anaesthesia.

It also remains unclear whether azaperone can be appropriately absorbed through the nasal mucous membranes. There are no studies available on this in pigs or other animal species. We oriented ourselves solely on the work of Axiak et al. [5]. It cannot be excluded that the lower efficiency of intranasally applied ketamine is partly due to a loss of efficacy of azaperone.

The prolonged recovery time after castration under ketamine anaesthesia is well described and accepted [2, 3, 10]. Axiak et al. [5] showed a way to significantly reduce this time by the application of the anaesthetic fluid intranasally. However, the efficiency of anaesthesia was also significantly reduced in their study. Axiak et al. [5] used a dosage of 15 mg kg^− 1^ body weight both, on the i.m. and the i.n. route. In the present study, we increased the dosage of S-ketamine to 30 mg kg^− 1^ for i.n. application. Although ketamine was not completely absorbed from the mucosa of the nasal cavity and thus, the effectivity of anaesthesia according to the movement scores and cortisol levels was not satisfying, the recovery time was as long as with i.m. ketamine application.

## Conclusions

The exchange of racemic ketamine by the pure S-enantiomer at the same dosage significantly reduced movement scores during castration of male piglets. However, at the same time, cortisol levels 120 min. after castration were significantly increased. Hypothermia reached the same level with S-ketamine than with racemic ketamine. As a major drawback, recovery time was significantly prolonged with S-ketamine as compared to racemic ketamine at the same dosage. Further studies should optimize the dose for S-ketamine in pigs using formulations higher concentrated. However, as even smaller effects on the movement scores during castration after i.n. application resulted in a prolonged recovery phase comparable with that induced by racemic ketamine i.m., S-ketamine does not seem to be an alternative for future anaesthesia in suckling piglets.

The intra-nasal application of S-ketamine reduced the applicability of ketamine anaesthesia to piglets. With the available formulation it was not possible to deliver enough of the drug intra-nasally. Additionally, the i.n. application of ketamine had no positive effect on the length of the recovery period. Thus, the application of ketamine by the i.n. route also does not seem to be an alternative for future anaesthesia in suckling piglets.

## Methods

This study was performed in accordance with established guidelines for the care and handling of laboratory animals and approved by the local Animal Welfare Authorities (Regierungspräsidium Giessen, Tierschutzkommission; permission no: *GI 18/15- No. 23-2014).*

### Animals

The experiment was performed on a practical piglet producing farm in Hesse. The piglets were housed in a typical conventional farrowing pen, with a piglet nest (temperature of more than 30 °C) and partially slatted floor. Sample size was determined to find a difference of castration score 1, with an alpha level of 0.05 and a power of 0.8. This required 13 litters with (at least) 6 male piglets each, i.e. a total of 78 animals. Seventy-eight male, healthy piglets from 13 litters were assigned to 6 groups at an age of 5 days. All piglets were clinically examined, weighed and ear-tacked. The piglets were all apparently healthy, had normal testicular anatomy, no malformation and no concurrent treatments. The complete procedure was carried out in a separate room close to the farrowing pens. The experiment was integrated into routine suckling pig castration, which was carried out in accordance with current legislation in Germany at the time of the test exactly as described for the positive control. All piglets lived unaffected after the end of the experiment.

### Experimental design

Piglets were matched by litter and weight to 6 groups (A-F) in a randomized and blinded experimental study (Table 3). Only litters with at least 6 healthy male piglets were used so that each piglet of a litter corresponded to a group and thus the piglets of different litters were not mixed and one litter was performed after the other. In litters with more than 6 male piglets, the supernumerary animals were treated like the females and not castrated during the experiment. Before weighting and randomization, the piglets were separated from the sow and all piglets of a litter were put into a warmed straw covered box. The piglets were only returned to the sow after all procedures described below had been completed, including castration and recovery phase. After randomization, the first blood sample was taken. Afterwards the NSAID (flunixin, Finadyne ®, Schering-Plough, France) was administered. Thirty min. after the NSAID administration, the first clinical examination took place. Then, depending on the group, the anaesthetic (ketamine-azaperone drug mixture) was injected. Fifteen min. later the surgical castration was performed. The time span of 15 min. is applied under practical conditions and was also chosen by Bettschart-Wolfensberger et al. [6] and Axiak et al. [5]. The fixed adherence to the time was important for standardizing the experiment. The piglets were all unconscious after 15 min. The degree of unconsciousness was clinically recorded shortly before castration (Table 4). Five and one hundred twenty minutes after castration, the second and third blood samples were collected, and the second and third clinical examination were conducted (Table 4). If piglets were not surgically castrated, they were handled in the same way as the castrated piglets (sham castrated). Anaesthesia and castration were performed by two different persons.

**Table 3:** Experimental design and treatments

Trial group	A	B	C	D	E	F
Treatment	15 mg kg^-1^** S-ketamine** + 2 mg kg^-1^ azaperone, i.m., surgical castration	15 mg kg^-1^** R/S-ketamine** + 2 mg kg^-1^ azaperone, i.m, surgical castration	30 mg kg^-1^** S- ketamine** +2 mg kg^-1^ azaperone, i.n., surgical castration	15 mg kg^-1^** R/S-ketamine** +2 mg kg^-1^ azaperone, i.m.; sham castrated	Positive control group; no anaesthesia, surgical castration^a^	Negative control group; no anaesthesia, sham castrated

*i.m.* intramuscular application, *i.n.* intranasal application of the anaesthetic

^a^piglets of trial group E were castrated according to the legal requirements in Germany at the time of the study. This procedure was necessary to fulfil the specifications of the local Animal Welfare Authorities. Piglets of all groups received an NSAID as presented below

**Table 4:** Schedule of treatments

Time point	Activity	Description
0 min.	Separation from sow, weighting and randomisation	
20 min.	1st blood sampling	Sample before castration/sham castration
20 min., 10 s	Application of NSAID	
30 min.	1st clinical examination	Examination (heart rate, breaths, rectal temperature) before castration/sham castration
35 min.	Application of Anaesthetics	
50 min.	Castration/sham castration	
55 min.	2nd blood sampling	Blood sample 5 min. after castration/sham castration (cortisol)
55 min.10 s	2nd clinical examination	Examination (heart rate, breaths, rectal temperature) 5 min. after castration/sham castration
170 min.	3rd blood sampling	Blood sample 120 min. after castration/sham castration (cortisol)
170 min. 10 s	3rd clinical examination	Examination (heart rate, breaths, rectal temperature) 120 min. after castration/sham castration

### Blood sampling and cortisol analysis

Blood samples were taken after randomization of the piglets, directly before the application of the NSAID, as well as 5 min. and 120 min. after castration by puncture of the Vena cava cranialis. One ml of blood was collected into a S-Monovette tube (Sarstedt, Germany). Samples were stored at 4 °C, serum was gained by standard centrifugation and frozen at -18 °C until further usage. Plasma cortisol concentrations were determined in ng/ml using an IMMULITE system (Siemens Diagnostic, USA). The intra-assay coefficient of variation was 6.3–10.0 % and the inter-assay coefficient of variation was 5.8–8.8 %.

### Analgesia

All 78 piglets within the study received the NSAID flunixin (Finadyne ®, Schering-Plough, France) at 2.2 mg kg^− 1^ i.m., 30 min. prior to castration [36] with a 0.4 × 40 mm needle (Braun, Melsungen, Germany) into the left neck muscle to alleviate suffering according to the present legislation in Germany. Flunixin was selected because an earlier study by the working group had shown a tendentious advantage of Flunixin over Metacam. However, the difference was not statistically significant [37].

### Clinical examination

All piglets were clinically examined at the three time points, thirty min. after application of NSAID, i.e. close to castration, 5 and 120 min. after castration. The clinical examination for the time points five and 12 min. after castration was performed after blood collection. This procedure was chosen to prevent direct effects on cortisol levels, although it was taken into account, that the blood sampling procedure was affecting clinical variables. Clinical variables were the pulse and respiratory rates and the rectal temperature.

### Anaesthesia

Intramuscular application of the R/S-ketamine racemate (15 mg kg^− 1^ body weight, Ursotamin®, Serumwerke Bernburg, Germany) or S-ketamine (15–30 mg kg^− 1^ body weight, Ketanest®, Pfizer, Germany: Esketaminhydrochloride), each in combination with azaperone (2 mg kg^− 1^ body weight, Stresnil ®, Elanco, Germany) was done with a 0.4 × 40 mm needle (Braun, Melsungen, Germany) into the right neck muscle. Intranasal application was partitioned into both nostrils with a LMA MAD Nasal™ applicator (Teleflex®, Germany). The piglets were held in a sternal position on the arm, with the nose upwards at a 45° angle. The nasal applicator was pressed on the nostril, sealing the room between applicator and nostril to minimize losses. According to the selected dosage of 30 mg kg^− 1^ S-ketamine and 2 mg kg^− 1^ azaperone, volumes of 1.6 to 4.0 ml were added, corresponding to a volume of 1.24ml kg^− 1^. The dose for the intranasal application had previously been tested in a preliminary trial under the same approval by the local Animal Welfare Authorities (Regierungspräsidium Giessen, Tierschutzkommission; permission no: GI 18/15- No. 23-2014). 30 mg S-ketamine/kg bodyweight was the highest volume dose that could be administered to the sucking piglets (data not shown). Nevertheless, uncontrolled losses of anaesthetic after the piglets were released could not be avoided. Piglets were left in a warmed, straw covered box to prevent hypothermia and to control the effect of anaesthesia. Piglets in controlled groups without anaesthesia (E and F) received 1.2 ml of a 0.9 % NaCl-solution (Braun, Melsungen, Germany) per nostril. Piglets were treated with castration or were sham castrated exactly 15 min. after injection.

### Castration

Castration was performed 15 min. after the application of ketamine/azaperone and NaCl, respectively. Piglets were fixed with the left hand, slightly pressing the testicals caudally. With the right hand a scalpel blade was used to apply two small incisions (1.5 cm) directly over the testes, parallel to the raphe scroti through the skin and the processus vaginalis. Testicles were then softly pulled through the incisions and the spermatic cord was cut with an emasculator (Hausmann, Eickemeier, Germany). The wound was treated with an iodine solution (Braun, Melsungen, Germany) and the piglets were immediately placed back to the warmed straw bedded box. The blade was disinfected between each piglet in a Povidone-iodine solution (WDT, Germany).

### Behavioural indices

Behavioural activities of the piglets were observed by video recording. Recording started with castration and continued during recovery phase until the last piglet of the litter was returned to the sow, 193 min. after recording began. Piglets were marked with large numbers on their backs for individual recognition and videos were live recorded. Movements during handling/castrations were scored from 0 to 4 (Table 1). Time spans from castration to first lifting of the head, first reaching of a sternal position, first standing and the time point, when piglets were returned to the sow were recorded. Piglets were returned to sow as soon as they could walk without staggering or falling over.

### Statistical analysis

Data were analysed with the program package IBM-SPSS, Version 27 (IBM, Munich, Germany). All variables were checked by QQ-plots, skewness and curtosis. The residues of all variables were found to be largely normally distributed. Analysis was done with a mixed effect linear model with the time point (before, 5 min. and 120 min. after castration/sham castration) and the study group as fixed effects and the piglet within litter as a random effect. The weight of the piglets was considered as a covariate. Results were presented as least square means with standard errors. Corrected coefficients of determination were additionally given. Significances were analysed pairwise with Bonferroni correction (α = 0.05).

## Data Availability

Data and materials are available from the authors on reasonable request.

## Abbreviations

°C: Grades Celsius
CNS: Central nervous system
i.m.: Intramuscular
i.n.: Intranasal
IU: International Units
i.v.: Intravenously
kg: Kilogram
mg: Milligram
ml: Milliliter
n. s.: Not significant
P: Probability
R^2^: Corrected coefficient of determination
